# Promoting the application of pediatric radiomics via an integrated medical engineering approach

**DOI:** 10.1002/cai2.44

**Published:** 2023-02-19

**Authors:** Haige Zheng, Fang Wang, Yang Li, Zhicheng Li, Xiaochun Zhang, Xuntao Yin

**Affiliations:** ^1^ Department of Radiology, Guangzhou Women and Children's Medical Center Guangdong Provincial Clinical Research Center for Child Health Guangzhou China; ^2^ Lianying Intelligent Medical Technology (Chengdu) Co., Ltd. Chengdu China; ^3^ Institute of Biomedical and Health Engineering, Shenzhen Institute of Advanced Technology Chinese Academy of Sciences Shenzhen China

**Keywords:** radiomics, pediatrics, oncology, survival prediction

## Abstract

Radiomics is widely used in adult tumors but has been rarely applied to the field of pediatrics. Promoting the application of radiomics in pediatric diseases, especially in the early diagnosis and stratified treatment of tumors, is of great value to the realization of the WHO 2030 “Global Initiative for Childhood Cancer.” This paper discusses the general characteristics of radiomics, the particularity of its application to pediatric diseases, and the current status and prospects of pediatric radiomics. Radiomics is a data‐driven science, and the combination of medicine and engineering plays a decisive role in improving data quality, data diversity, and sample size. Compared with adult radiomics, pediatric radiomics is significantly different in data type, disease spectrum, disease staging, and progression. Some progress has been made in the identification, classification, stratification, survival prediction, and prognosis of tumor diseases. In the future, big data applications from multiple centers and cross‐talent training should be strengthened to improve the benefits for clinical workers and children.

## Graphical abstract

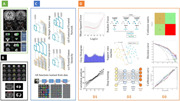

**Abbreviations**
AT/RT: atypical teratoid/rhabdoid tumor
CNN: convolutional neural network
DMGs: diffuse midline gliomas
DT: decision tree
EFS: event free survival
EP: ependymoma
GLCM: gray level co‐incidence matrix texture
GLDM: gray level dependence matrix
GLRLM: gray level run length matrix
GLSZM: gray level size zone matrix
HB: hepatoblastoma
ICPP: idiopathic central precocious puberty
LASSO: least absolute shrinkage and selection operator
LR: logistic regression
MB: medulloblastoma
MRI: magnetic resonance imaging
NGTDM: neighboring gray tone difference matrix
NTM: nontuberculous mycobacterial
OS: overall survival
PA: pilocytic astrocytoma
RF: random forest
RMS: rhabdomyosarcoma
ROI: region of interest
WT: Wilms tumor
YST: yolk sac neoplasm

## 1 INTRODUCTION

Radiomics research uses automated algorithms to extract a large number of features that cannot be identified by humans from visual inspection of medical images. This machine‐based approach combines diversified statistical analysis and data‐mining methods to find the key information conducive to the research objectives. This information is ultimately used to assist in the diagnosis, classification, grading, and prognostic prediction of diseases. Radiomics is widely used in adult tumors, but there are few reports in pediatrics. The application of radiomic techniques to pediatric diseases, especially for neoplastic lesions, might be a new area of great promise. According to statistics, about 429,000 children and adolescents are newly diagnosed with cancer every year in the world. The survival rate of children with cancer in low‐ and middle‐income countries is significantly lower than that in developed countries [1]. Early diagnosis and stratified treatment are key to achieving WHO's 2030 “Global Initiative on Childhood Cancer,” and radiomics carries unique advantages in this regard. Based on a PubMed database search with the keyword “radiomics,” a total of more than 6800 related papers were retrieved for the time period between 2015 and 2022. When the search formula was set as “((child) OR (pediatrics) OR (children)) AND (radiomics),” 330 radiomics papers related to pediatric diseases were retrieved, accounting for less than 5% of the total radiomics papers. The number of radiomic research papers has increased dramatically in recent years, indicating that this field has drawn substantial attention from the research community.

## 2 THE NATURE OF RADIOMICS AND ITS APPLICATION IN THE FIELD OF PEDIATRICS

### 2.1 General characteristics of radiomics

**Figure 1:**
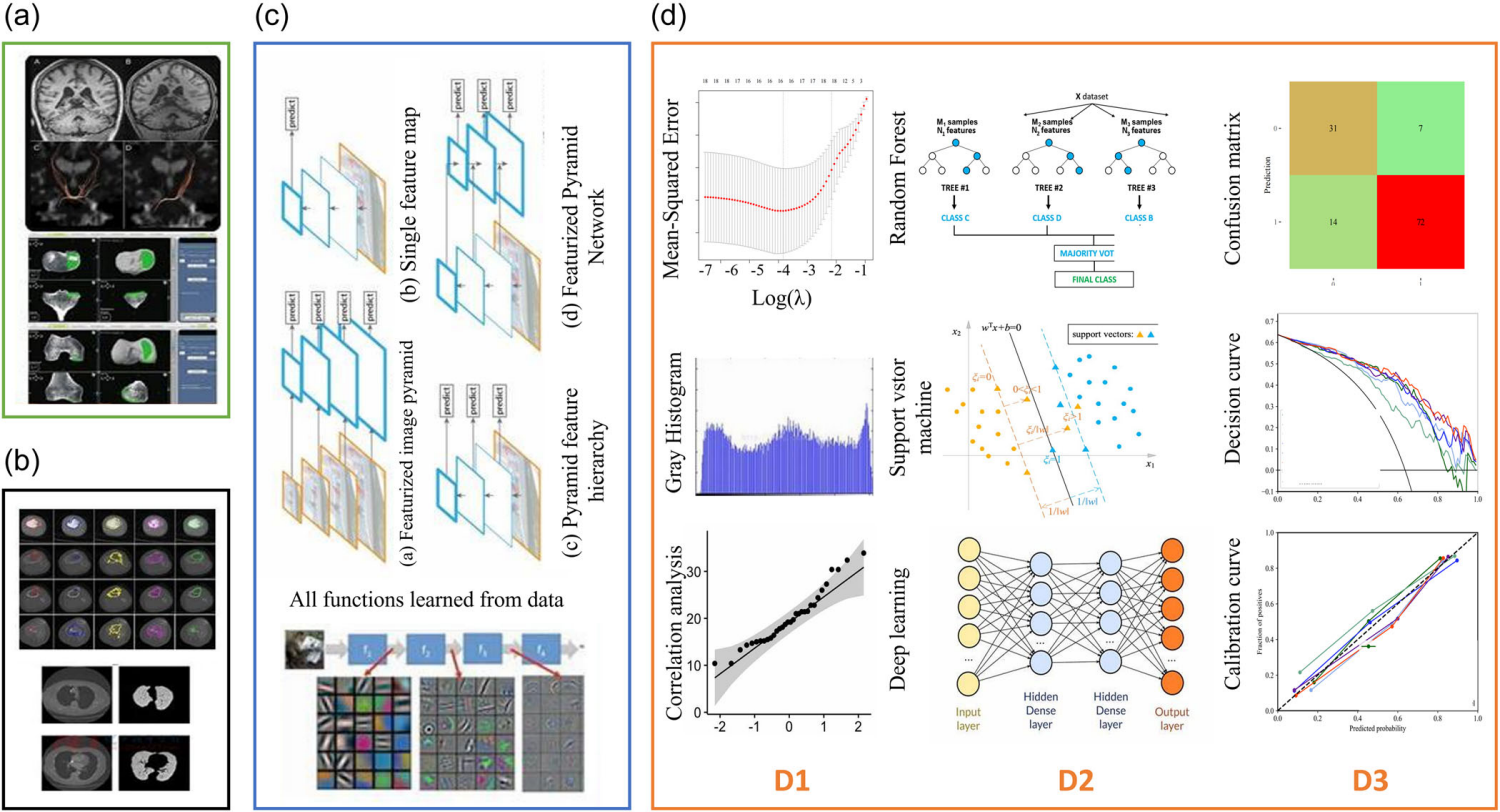
Radiomics analysis flow chart. (a) Image acquisition and reconstruction: Original images were acquired from CT, MRI, and other equipment, and 3D reconstruction was performed on the original images. (b) Image segmentation: using automatic, semiautomatic, or manual methods, the region of interest (ROI) of the acquired original image is delineated to obtain the data source. (c) Feature extraction: There are various methods of feature extraction, but all of them are learned from the hidden numbers behind the images. Through the implementation of the algorithm, thousands of features, including shape‐based features, first‐order statistics features, texture features, and wavelet features, can be extracted from the ROI region delineated in image segmentation. (d) Model building: including feature selection (LASSO regression, variance threshold, Pearson's correlation coefficient, etc.), model construction (random forest, support vector machine, Neural network, XGBoost, etc.), model validation (area under receiver characteristic curve, decision curve, calibration curve, etc.). CT, computed tomography; LASSO, least absolute shrinkage and selection operator; MRI, magnetic resonance imaging.

Radiomics research usually includes five steps: data collection, region of interest (ROI) delineation, feature extraction, feature screening, model construction, and validation (Figure 1). After data collection, data should be standardized according to data sources. FAIR (findability, accessibility, interoperability, and reuse) data criteria provide standardized guidelines for radiomics research [2]. At the same time, potential confounding factors should be controlled during data processing to ensure the reliability of the research conclusions [3]. Because of the intrinsic degree of subjectivity associated with artificial ROI delineation, radiomic features with high stability and reproducibility should be further investigated via a combination of within‐group and between‐group correlation coefficient analyses [4].

**Figure 2:**
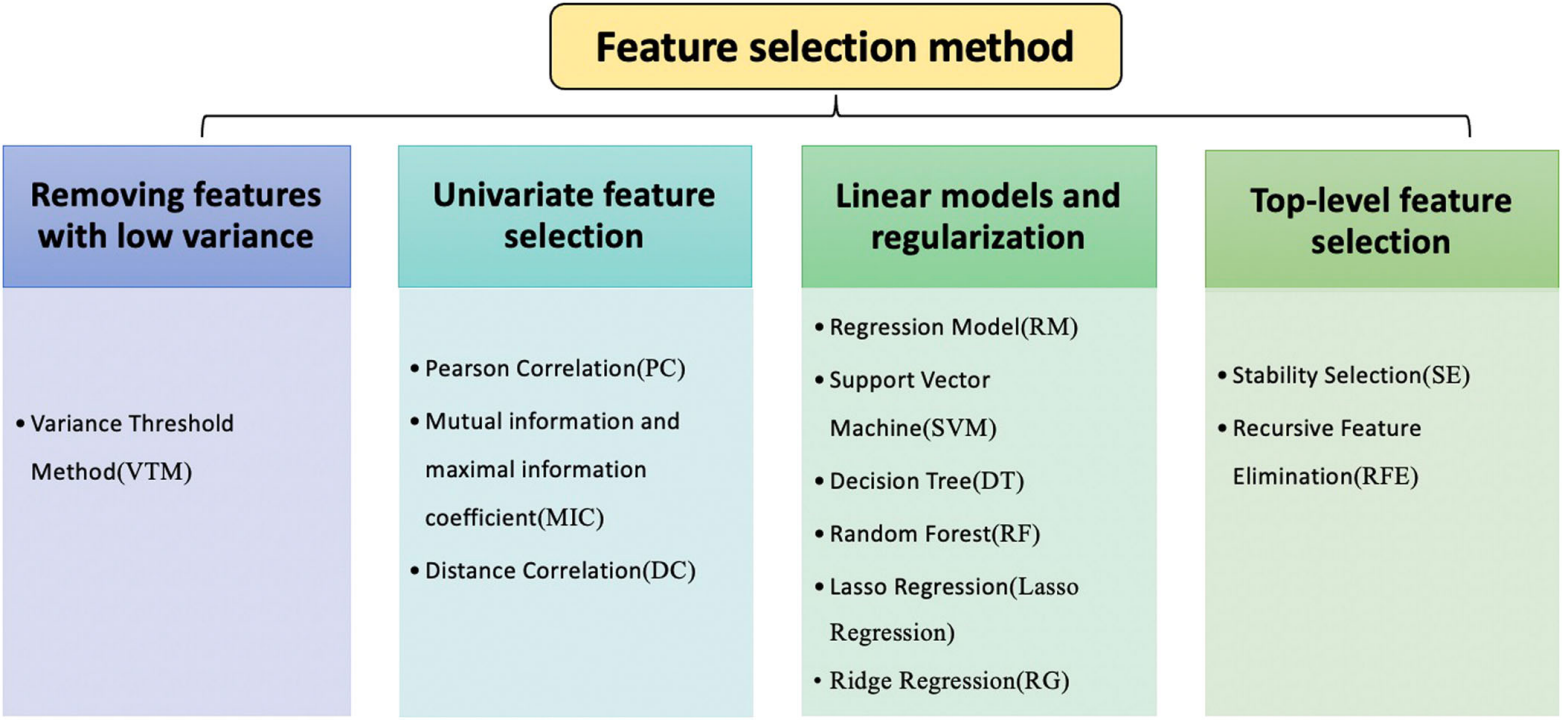
Classification of feature selection methods

**Figure 3:**
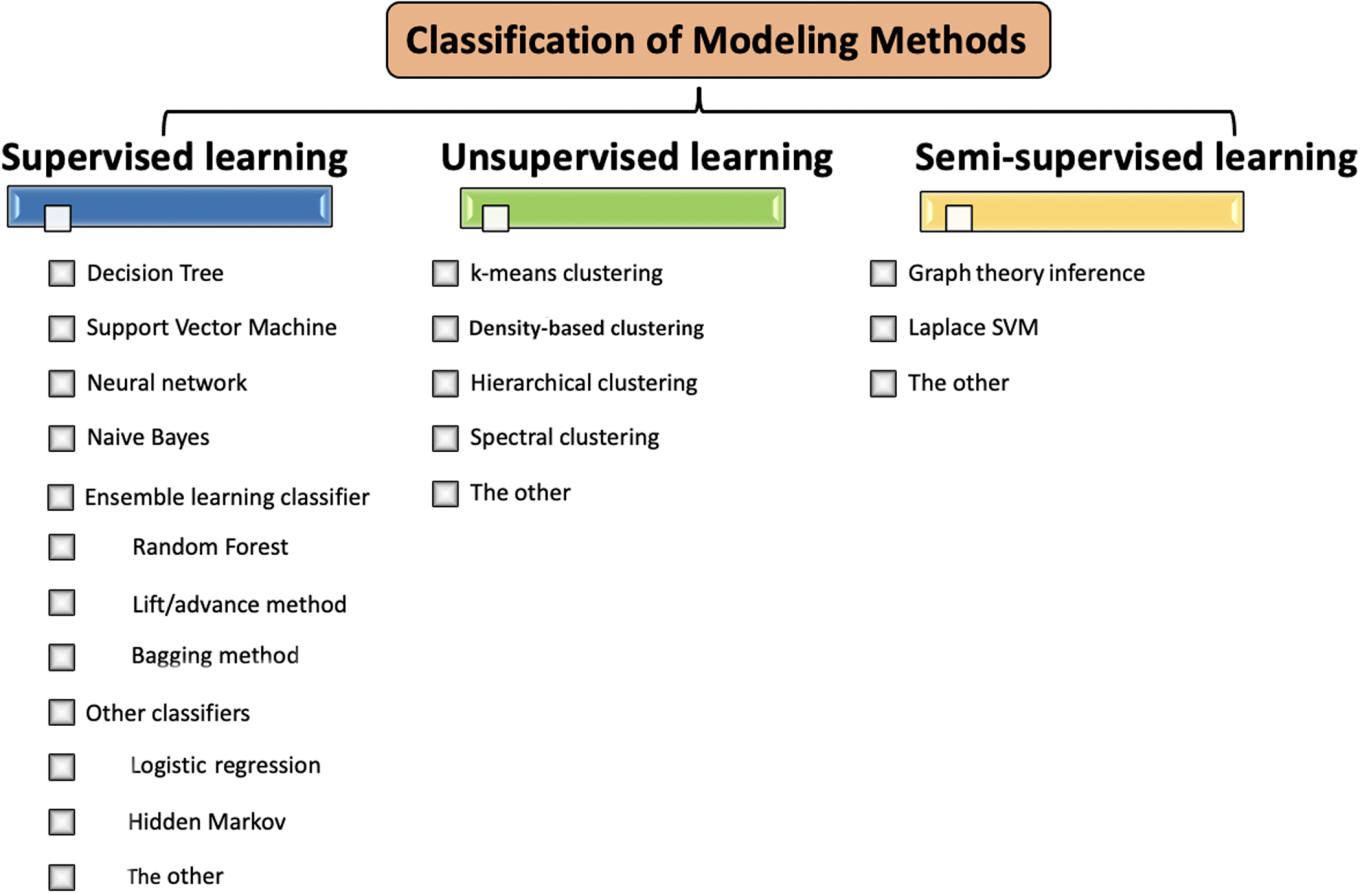
Classification of modeling methods

Feature screening methods can be divided into two categories: feature selection and feature dimension reduction. The result of feature selection is derived from the original feature set, so it is highly interpretable. Feature screening methods can be divided into four groups: removal of features with low variance, univariate feature selection, linear model and regularization, and two top‐level feature selection algorithms (Figure 2). These four methodological approaches are usually combined for feature selection, and cross‐validation is used to preserve features with high stability for building a more robust prediction model [5]. Depending on the target problem, different modeling methods can be adopted. These methods can be divided into three main categories (Figure 3): supervised learning, unsupervised learning, and semisupervised learning [6, 7]. In radiomics, different algorithms will affect the prediction performance of the associated model. Therefore, a variety of modeling methods should be selected for relevant research. Common terms related to radiomics are explained in Table 1.

**Table 1:** Explanation of common terms related to radiomics

Terms related to radiomics	Description
Radiomics	An auxiliary clinical decision‐making approach in which high‐throughput image cluster features are extracted from computed tomography (CT), magnetic resonance imaging (MRI), and PET/CT or ultrasound medical images and comprehensively analyzed quantitatively to improve diagnostic, prognostic, and predictive accuracy
Shape‐based features	The radiomics features that describe the shape of the lesion area, including voxel volume, maximum 3D diameter, mesh volume, major axis length, sphericity, minimum axis length, elongation, surface volume ratio and so forth
First‐order statistics features	The radiomics features that describe the distribution of voxel values in an image using common and basic algorithms, including the maximum, minimum, mean, median, range, energy, entropy, and the histogram kurtosis and skewness
Texture‐based features	The radiomics features that reflects the spatial distribution relationship of voxel intensities between voxels, including gray level cooccurence matrix (GLCM), gray level dependence matrix (GLDM), gray level run length matrix (GLRLM), gray level size zone matrix (GLSZM), neighboring gray tone difference matrix (NGTDM)
Wavelet features	A kind of complex feature obtained by transforming the original image with wavelet filter
Least absolute shrinkage and selection operator (LASSO)	A regression method mainly used for variable selection, which reduces insignificant feature coefficients to zero by adjusting the size of the penalty parameter *λ*, that is, removing insignificant features
Decision tree (DT)	A basic classification and regression method that typically recursively selects the best feature and splits according to that feature
Random forest (RF)	A supervised machine learning algorithm built from decision tree algorithms for solving classification and regression problems. The forest composed of classification trees is called a random forest classifier, and the forest integrated by regression trees is called a random forest regressor
Support vector machine (SVM)	A kind of generalized linear classifier that classifies data bivariate according to supervised learning. It uses a loss function to calculate the empirical risk and adds a regularization term to the solution system to optimize the structural risk, which is a sparse and robust classifier
Convolutional neural network (CNN)	A special feedforward neural network for learning hierarchical representations of image data that operate directly on the original image. The convolutional layer and pooling layer are used to reduce the dimensionality of image features and try to extract high‐representation image features automatically
Logistic regression (LR)	A generalized linear regression suitable for regression analysis, often used in data mining, disease diagnosis, prediction and other fields. Because of its simplicity, it has become the most popular and commonly used supervised classifier

Radiomics research is a data‐driven science. Data quality, data diversity, and sample size are key factors that determine the performance of the final model. Successful radiomic applications require expert knowledge in many related areas, such as clinical medicine, imaging, statistics, computer science, and machine learning, which poses numerous challenges for radiomic researchers [8]. It is, therefore very difficult to complete the whole analysis process by relying only on the individual contribution of doctors, making it necessary to combine medicine with engineering. At present, there are few established medical and engineering interdisciplinary research teams in China, and many of them are dominated by engineering scholars, such as teams at the Beihang University Medical and Engineering Interdisciplinary Innovation Research Institute or those at the Xi'an Jiaotong University Medical and Engineering Interdisciplinary Research Institute. Medical experts usually “package” data and hand it over to university or enterprise personnel for data processing. This “stitched” crossover approach limits the clinical promotion and commercial value of radiomics. Such difficulties exist not only for pediatric radiomic researchers but for all radiomic researchers. Bridging the gap between clinical needs and the technological development of radiomics will require the establishment of truly interdisciplinary research and translational platforms, and the training of “native” talents with backgrounds in both medicine and engineering.

### 2.2 The particularity of pediatric radiomics

In terms of data acquisition, children present additional difficulties during radiological examination because of their poor compliance. At the same time, because children are more sensitive to radiological examination and radiation exposure than adults, image data mostly depend on ultrasound and magnetic resonance imaging (MRI) rather than computed tomography (CT) [9]. In addition, the sequences used in MRI radiomics for children are mostly based on conventional sequences, such as T1‐weighted images (T1WI), T2‐weighted images (T2WI), fluid‐attenuated inversion recovery images, and diffusion‐weighted images. Advanced functional sequences, such as cerebral perfusion imaging and spectral sequences, have been primarily restricted to adult radiomic studies [10]. One possible explanation is that more advanced sequences are specifically designed for tumors in adults, which are more common in clinical practice. For all these reasons, from the perspective of raw data collection, there are some important differences between pediatric and adult radiomics.

Differences between the types of radiomics include the fact that tumors in adults are dominated by somatic mutations, while pediatric tumors are mainly caused by germline mutations. These two tumor classes are associated with different therapeutic effects and prognostic ability [11]. For example, common tumors in children, such as neuroblastoma and rhabdomyosarcoma, are rare in adults, so findings from adult radiomics cannot be simply generalized to pediatric diseases. The radiomic features extracted from pediatric radiomics are simpler than those obtained from adults, and texture features or wavelet features are rarely extracted from children [12]. Compared with adults, the research interests of pediatric radiomics are relatively limited. Currently, pediatric radiomics is mainly applied to oncological diagnosis, with only a few studies focusing on the association with genotypes or pathological findings, the evaluation of therapeutic effects, and the prediction of disease progression. In addition, staging criteria for pediatric tumors vary by category of disease, evolve over time, and usually lack standardized guidelines [13]. Therefore, there are fundamental differences between radiomic research in children and adults.

Accurate diagnosis has always been a difficult problem in pediatric imaging. Multicenter cooperation can solve the problem of limited sample size and increase the diversity of data sources, which can enhance the generalization performance of the adopted model. However, because of constraints associated with medical data privacy and institutional equity allocation, multicenter data is rarely suitable for specific machine learning applications. The emergence of federated learning has offered a potential technical solution for addressing the limitations of multicenter research. However, it remains unclear how long it will take before federated learning comes into practice [14].

## 3 APPLICATION STATUS OF RADIOMICS IN PEDIATRIC DISEASES

Children undergo rapid development of systemic tissues and organs, with their physiological and psychological states constantly improving. Radiomic technology provides valuable quantitative information for differential diagnosis, classification, and prognostic prediction of pediatric diseases. Further, future trends will associate radiomic information with genetic information about pediatric diseases, thus revealing gene function and establishing a relationship between the organism's genome and the associated phenotype. In view of the above considerations, we reviewed relevant literature from the past three years (Table 2).

**Table 2:** Literature related to pediatric radiomics

Studies	Published time	Patients	Number of patients	Target type	Image type
Li et al. [15]	2020	EP, PA	45	Differential diagnosis of tumor	MRI
Dong et al. [16]	2021	EP, MB	51	Differential diagnosis of tumor	MRI
Chen et al. [17]	2020	RMS, YST	94	Differential diagnosis of tumor	CT
Zhang et al. [18]	2021	AT/RT, MB	144	Differential diagnosis of tumor	MRI
Ma et al. [19]	2022	WT	118	Tumor grading and classification	CT
Chang et al. [20]	2021	MB	38	Tumor grading and classification	MRI
Giraudo et al. [21]	2022	Pediatric soft tissue sarcomas	18	Tumor grading and classification	MRI
Tam et al. [22]	2021	DMGs	177	Prognostic prediction	MRI
Wagner et al. [23]	2022	DMGs	100	Prognostic prediction	PET/MR
Jiang et al. [24]	2021	HB	88	Prognostic prediction	CT
Zhang et al. [25]	2021	EP	157	Prognostic prediction	MRI
Lin et al. [26]	2022	Osteosarcoma	191	Prognostic prediction	CT
Jiang et al. [27]	2021	ICPP	30	Evaluation of idiopathic central precocious puberty	MRI
Shin et al. [28]	2021	Premature infant	46	Prediction of psychomotor dysfunction	MRI
Xuan et al. [29]	2021	Pregnant women with or without placental invasion	352	Prenatal prediction of placental infiltration	MRI
Bulushi et al. [30]	2022	Lymphadenopathy	142	Distinguish NTM lymphadenitis from other lymphadenopathy	CT

Abbreviations: AT/RT, atypical teratoid/rhabdoid tumor; CT, computed tomography; DMGs, diffuse midline gliomas; EP, ependymoma; HB, hepatoblastoma; ICPP, idiopathic central precocious puberty; MB, medulloblastoma; MRI, magnetic resonance imaging; NTM, nontuberculous mycobacterial; PA, pilocytic astrocytoma; RMS, rhabdomyosarcoma; WT, Wilms tumor; YST, yolk sac neoplasm.

### 3.1 Differential diagnosis of tumors

Early studies focused on the differential diagnosis of posterior fossa tumors. Li et al. [15] explored differences in MRI radiomic characteristics between ependymoma (EP) and pilocytic astrocytoma (PA) based on imaging data from 45 children with posterior fossa tumors. The results showed that MRI radiomics can quantitatively and objectively assess the internal heterogeneity of EP and PA, and can successfully distinguish them before surgery. In related research, Dong et al. [16] collected T1WI and apparent diffusion coefficient maps from 24 EP and 27 MB children. These authors used semiautomatic segmentation methods to delineate 3D tumor regions, and they extracted radiomic features to distinguish EP and MB in childhood.

In addition, Chen et al. [17] retrospectively studied CT images from 49 cases of pelvic rhabdomyosarcoma (RMS) and 45 cases of yolk sac neoplasm (YST) in children. These authors extracted radiomic features based on the plain scan, arterial phase, and venous phase. Using these features, they constructed a radiomic model to distinguish between RMS and YST. Atypical teratoid/rhabdoid tumors (AT/RT) and MB present similar imaging and histological features. Zhang et al. [18] collected T2WI and gadolinium‐enhanced T1WI (T1WI+C) images from 48 children with AT/RT and 96 children with MB from seven institutions. By relying on five radiomic features for distinguishing between AT/RT and MB children, their logistic regression model reached an area under the curve (AUC) value of 0.86 when applied to an independent test set.

### 3.2 Tumor grading and classification

Because treatment and prognosis differ for different tumor stages and molecular subtypes, it is necessary to accurately stage and classify tumors. To address tumor grading, Ma et al. [19] retrospectively analyzed CT images from 118 children with pathologically confirmed Wilms tumor (WT), and identified the staging of pediatric WT via radiomic technology before surgery. In this study, radiomic features were extracted and screened from CT‐enhanced portal vein phase images, and a support vector machine was used to develop the prediction model. This research showed that the radiomic model based on the portal phase of CT images can accurately predict the staging of WT in pediatric patients before surgery.

It is well‐known that different molecular subsets of MB in children differ significantly with respect to prognosis and treatment. Chang et al. [20] established a comprehensive prediction model of radiomics and clinical information based on MRI images and clinical data from 38 MB children. These authors used their model to achieve accurate prediction of molecular MB subtypes in children. Their results showed that eight radiomic gray level co‐incidence matrix texture features were significantly different among the four molecular subgroups of MB. Subsequently, Giraudo et al. [21] presented the first PET/MR‐based application of radiomics to pediatric soft tissue sarcoma. They demonstrated that the radiomic features extracted from the T2W sequence may act as biomarkers for distinguishing pediatric soft tissue sarcomas of different grades and histotypes.

### 3.3 Prediction of tumor prognosis

In terms of prognostic prediction, the entry point of pediatric radiomics is mainly focused on pediatric tumors, including diffuse midline gliomas (DMGs), hepatoblastoma, fossa EP, and advanced osteosarcoma. DMGs are fatal pediatric brain tumors. In two studies, Tam et al. [22] and Wagner et al. [23] collected MRI images from hundreds of untreated children with DMGs, extracted relevant radiomic features and established a model to predict their overall survival (OS) or progression‐free survival. The results showed that it was feasible to evaluate the prognosis and survival rate of patients with DMGs via radiomic methods. Using the same analysis method, Jiang et al. [24] collected portal vein CT images from 88 children with hepatoblastoma confirmed by histology from two institutions and extracted relevant radiomic features. A radiomic score of event‐free survival (EFS) was constructed by integrating the least absolute shrinkage and selection operator (LASSO)‐Cox model, and the EFS Cox regression model was established by incorporating other clinical and radiological data. The results showed that radiomic characteristics could be used as a prognostic indicator for children with hepatoblastoma.

The recurrent risk of posterior fossa EP largely depends on styles of surgical operation and molecular status (posterior fossa ependymoma A, PFA; posterior fossa ependymoma B, PFB). A previous study extracted 1800 quantitative features from preoperative T2WI and T1WI+C images from 157 patients with posterior fossa EP and submitted these features to a Cox proportional hazards regression model for survival analysis. The results showed that radiomic features based on T1WI+C could effectively distinguish between PFA and PFB (AUC = 0.86), and that the risk score based on T2WI radiomic features could significantly distinguish between the high‐risk group and the low‐risk group [25].

Another study [26] retrospectively analyzed CT images from 191 patients with advanced osteosarcoma who had received neoadjuvant chemotherapy. These authors used within‐group and between‐group correlation coefficients and correlation analysis to select robust features, and further constructed the radiomic label using the LASSO algorithm. They employed a multi‐factor logistic regression model to construct an individualized pathological response evaluation model that incorporated radiomic labels and clinical information before neoadjuvant chemotherapy.

### 3.4 Nontumor research

Since its introduction in 2012, radiomics research has extended beyond the scope of cancer. Researchers have explored the application of radiomics to other nontumor diseases, such as growing development, premature infants, pregnancy‐related diseases, and inflammatory lesions in children.

For example, the traditional method of measuring pituitary height and volume via MRI is time‐consuming. Therefore, researchers [27] have attempted to objectively and quantitatively evaluate idiopathic central precocious puberty (ICPP) using methods based on MRI texture analysis. This study collected data from 12 healthy girls and 18 ICPP girls. Texture features were extracted from the pituitary gland, and the LASSO‐linear regression method was used to establish the ICPP prediction model. The results showed that, compared with traditional manual measurements of pituitary height (AUC = 0.68), radiomic labels showed better predictive performance (AUC = 0.76).

Shin et al. [28] prospectively enrolled 46 preterm infants who underwent brain MRI at or near full‐term age. They then applied radiomic analysis to white matter regions from T1WI and T2WI with five‐fold cross‐validation. Their model for predicting psychomotor dysfunction in preterm infants from T1WI, T2WI, and T1WI, combined with T2WI, produced AUC values of 0.93, 0.83, and 0.90, respectively. Xuan et al. [29] retrospectively included abdominal MRI data from 352 pregnant women (147 cases without placental invasion and 205 cases with placental invasion). These authors used a U‐net to segment placental tissue from abdominal MRI images and extracted relevant radiomic features from the resulting ROI to construct a radiomic model for prenatal diagnosis of placental infiltration. In addition, Al Bulushi et al. [30] applied radiomics to the diagnosis of lymphadenitis. Their results showed that radiomic features generated by a random forest classifier could distinguish between NTM lymphadenitis and other causes of lymphadenopathy, with an accuracy value of 82% and an AUC of 89%.

## 4 SUMMARY AND PROSPECT

Pediatric radiomics research usually includes five steps: data collection, ROI delineation, feature extraction, feature screening, model construction, and validation. Common tools for mapping ROI include ITK‐SNAP (www.itksnap.org), 3D‐Slicer (www.slicer.org), and MaZda (http://eletel.eu/mazda). Feature extraction software includes MaZda (http://eletel.eu/mazda), 3DSlicer (www.slicer.org), and IBEX (http://bit.ly/IBEX_MDAnderson). Software such as R (www.r-project.org/) and Python (www.python.org/) is often used to analyze radiomic features.

At present, MRI images are widely used in pediatric radiomics research, but study sequences for pediatric MRI radiomics are relatively simple and are mostly based on routine sequences. Therefore, functional sequences should be considered in the future [10]. In addition, pediatric radiomics should include diversified radiomic features, such as texture features and wavelet features. Of the pediatric radiomics studies reviewed above, only six carried a sample size of more than 100 cases, and one study had a sample size of more than 200 cases. The sample size of children enrolled in imaging studies is relatively small, and validation between different research centers is lacking. Although there are several public databases focusing on imaging studies, such as TCIA (https://www.cancerimagingarchive.net/), MedPix (https://medpix.nlm.nih.gov/home), and Grand Challenges (https://grand-challenge.org/), most databases involve adults, with only a few examples of tumors in children. The creation of pediatric databases should be encouraged in the future. At the present stage, researchers should adopt K‐fold cross‐validation for feature selection and model establishment when the sample size is limited, and should rely on prospective or multicenter data collection for independent testing. Unless these precautions are taken, the experimental conclusions might fail to generalize to other settings. In the future, it will be important to further improve data management, data preprocessing, experimental design, statistical processing, multicenter cooperation, and the creation of interdisciplinary talent training in the field of pediatric radiomics.

## AUTHOR CONTRIBUTIONS

**Haige Zheng**: Formal analysis (equal); methodology (equal); software (equal); validation (equal); writing – original draft (lead). **Fang Wang**: Formal analysis (equal); project administration (equal); software (equal); validation (equal). **Yang Li**: Formal analysis (equal); investigation (equal). **Zhicheng Li**: Formal analysis (equal); investigation (equal). **Xiaochun Zhang**: Supervision (equal); writing – review and editing (equal). **Xuntao Yin**: Supervision (equal); validation (equal); writing – review and editing (lead).

## CONFLICT OF INTEREST

Professor Xuntao Yin is the member of the *Cancer Innovation* Editorial Board. To minimize bias, he was excluded from all editorial decision‐making related to the acceptance of this article for publication. The remaining authors declare no conflict of interest.

## ETHICS STATEMENT

Not applicable.

## INFORMED CONSENT

Not applicable.

## Data Availability

Not applicable.
